# Item analysis: the impact of distractor efficiency on the difficulty index and discrimination power of multiple-choice items

**DOI:** 10.1186/s12909-024-05433-y

**Published:** 2024-04-24

**Authors:** Assad Ali Rezigalla, Ali Mohammed Elhassan Seid Ahmed Eleragi, Amar Babikir Elhussein, Jaber Alfaifi, Mushabab A. ALGhamdi, Ahmed Y. Al Ameer, Amar Ibrahim Omer Yahia, Osama A. Mohammed, Masoud Ishag Elkhalifa Adam

**Affiliations:** 1 Department of Anatomy, College of Medicine, University of Bisha, 61922 Bisha, Saudi Arabia; 2 Department of Microbiology, College of Medicine, University of Bisha, 61922 Bisha, Saudi Arabia; 3 Department of Biochemistry College of Medicine, Nile University, Khartoum, Sudan; 4 Department of Child Health, College of Medicine, University of Bisha, 61922 Bisha, Saudi Arabia; 5 Department of Internal Medicine, College of Medicine, University of Bisha, 61922 Bisha, Saudi Arabia; 6 Department of Surgery, College of Medicine, University of Bisha, 61922 Bisha, Saudi Arabia; 7 Department of Pathology, College of Medicine, University of Bisha, 61922 Bisha, Saudi Arabia; 8 Department of Pharmacology, College of Medicine, University of Bisha, 61922 Bisha, Saudi Arabia

**Keywords:** Psychometric, Items, Analysis, Distractor efficiency, Sifficulty index, Discrimination index, Correlation

## Abstract

**Background:**

Distractor efficiency (DE) of multiple-choice questions (MCQs) responses is a component of the psychometric analysis used by the examiners to evaluate the distractors’ credibility and functionality. This study was conducted to evaluate the impact of the DE on the difficulty and discrimination indices.

**Methods:**

This cross-sectional study was conducted from April to June 2023. It utilizes the final exam of the Principles of Diseases Course with 45 s-year students. The exam consisted of 60 type A MCQs. Item analysis (IA) was generated to evaluate KR20, difficulty index (DIF), discrimination index (DIS), and distractor efficiency (DE). DIF was calculated as the percentage of examinees who scored the item correctly. DIS is an item’s ability to discriminate between higher and lower 27% of examinees. For DE, any distractor selected by less than 5% is considered nonfunctional, and items were classified according to the non-functional distractors. The correlation and significance of variance between DIF, DI, and DE were evaluated.

**Results:**

The total number of examinees was 45. The KR-20 of the exam was 0.91. The mean (M), and standard deviation (SD) of the DIF of the exam was 37.5(19.1), and the majority (69.5%) were of acceptable difficulty. The M (SD) of the DIS was 0.46 (0.22), which is excellent. Most items were excellent in discrimination (69.5%), only two were not discriminating (13.6%), and the rest were of acceptable power (16.9%). Items with excellent and good efficiency represent 37.3% each, while only 3.4% were of poor efficiency. The correlation between DE and DIF (*p* = 0.000, *r*= -0.548) indicates that items with efficient distractors (low number of NFD) are associated with those having a low difficulty index (difficult items) and vice versa. The correlation between DE and DIS is significantly negative (*P* = 0.0476, *r*=-0.259). In such a correlation, items with efficient distractors are associated with low-discriminating items.

**Conclusions:**

There is a significant moderate negative correlation between DE and DIF (*P* = 0.00, *r* = -0.548) and a significant weak negative correlation between DE and DIS (*P* = 0.0476, *r* = -0.259). DIF has a non-significant negative correlation with DIS (*P* = 0.7124, *r* = -0.0492). DE impacts both DIF and DIS. Items with efficient distractors (low number of NFD) are associated with those having a low difficulty index (difficult items) and discriminating items. Improving the quality of DE will decrease the number of NFDs and result in items with acceptable levels of difficulty index and discrimination power.

## Background

High-quality MCQs are considered an appropriate assessment tool because they can cover a wide range of knowledge and domains of knowledge [1]. Many authors reported the validity and reliability of MCQs [2–6]. The validity and reliability of MCQs can be ensured pre-construction by the presence of the content material from which the MCQs will be constructed and a blueprint [7–10]. Item analysis is a post-examination method for ensuring the validity and reliability of MCQs [11]. It provides feedback to tutors about the constructed items and the coverage of the content materials from which items were created [12–14]. IA is a mathematical analysis of the examinee’s responses on an examination or test [3, 13]. Item analysis parameters include KR20, difficulty index (DIF), discriminating index (DIS), and distractor efficiency (DE) [4, 15]. Many authors reported that the exam quality depends on items’ difficulty and their discrimination index (power) [4, 16, 17]. For the ideal balanced exam, it was advised that 5% of the exam items could be easy, 20% moderately easy, 20% moderately difficult, 5% difficult items, and 50% for average ones [18, 19].

Type A MCQs are made of a stem that may have a leading question followed by four or three distractors and one key answer [4]. Distractors should appear similar to the key answer and convey a miss concept about the key (best) answer. Technically, distractors should be homogenous and devoid of grammatical and style errors [3, 20, 21]. The DE is the ability of distractors to distract the students from the key answer [22]. A functioning distractor (FD) can distract students from the key answer and is selected by more than 5% of the examinees [23, 24]. Any option chosen by less than 5% of the examinee is counted as a non-functional distractor (NFD) [24]. Because NFD can be identified and eliminated easily by all students, it makes items easier, impacts its discrimination power, and will have low efficiency [3, 15, 22, 25, 26].

Many studies and research work discussed the relationship between the different parameters of item analysis such as difficulty index, discrimination index, and exam reliability, as well as their relation and impact on each other [27–30]. The research work about DE is less. There is a gap in knowledge about the relation between DE and other parameters of item analysis. Also, there is a gap in knowledge about how DE impacts exam reliability, discrimination index, and difficulty index. NFDs in items have many causes, such as defective construction and low cognitive levels, mastering the content material from which the items were constructed, or repeated use of the item [31]. Some causes of NFD are related to item constructors and others to curriculum and blueprinting. Shedding light on the effect of DE on exam reliability, difficulty index, and discrimination index will stimulate more focus on the training of item constructors, curriculum mapping, and the importance of blueprinting.

This study was conducted to evaluate the impact of the DE on the difficulty and discrimination indices. The study findings and discussion will benefit academics interested in educational assessment, curriculum design, and mapping.

## Methods

### Study design and sampling

The study design is a cross-sectional, analytic study [32]. It was conducted at the College of Medicine, University of Bisha, from April to June 2023. The sampling technique covers the total coverage of registered students in level two on the course of the principle of human diseases.

The study population is students in level two in the College of Medicine. The students were from the annual university intake and studied the same curriculum in secondary school and first year at the College of Medicine. Thus, the study group is considered homogenous, and differences among them were considered due to their abilities and responses to items.

### Study context

The study utilized a standard item (psychometric) analysis of the final exam of the principle of human diseases course. The course is an integrated, multidisciplinary implemented in semester two of the second year. The total number of registered students on the course is 45. The students represent one patch taught by the same staff members in the educational environment. The total number of evaluated exam papers was 45. The student’s age and GPA were obtained from the student’s registration office.

The course’s final exam comprised 60 items (type A MCQs). Each item is formed of a stem followed by three distractors and a single best answer. Following the exam, the student’s answer sheets were checked, verified, and scanned by Apperson, Data Link 1200 scanner. On exam marking, there is no penalty for blank or wrong answers. The exam scanner provides a standard item analysis obtained and processed for the study.

### Calculation parameters of item analysis

Item DIF (easiness, P-value of item, absolute difficulty) is calculated as the percentage of examinees who score the item correctly. The value of DIF ranges from 0 to 100%. Items with DIF ≥ 78% are considered easy, items in the 78–25% range are acceptable and those less than 25% are difficult [14, 28, 33]. DIS is an item’s ability to discriminate between higher and lower (27%) achievers in the concerned item. The value of DIS ranges from − 1.00 to + 1.00. Negative items are non-discriminating, while the positives are discriminating. The discriminating items are categorized as poor (≤ 20), acceptable (0.21 to 0.24), good (0.25–0.34), and excellent (≥ 0.35) discriminating [14, 28, 33, 34]. The DE assesses the credibility of the items’ distractors to distract the examinee from the best answer. Each distracter selected by more than 5% of the examinees is considered a functioning distractor (FD), and those chosen by less than 5% are considered non-functioning distractors (NFD). Items are classified according to the numbers of NFDs to excellent (NFDs = 0), good (NFDs = 1), acceptable (NFDs = 2), and poor (NFDs = 3) [3, 15, 22, 28, 33, 35].

### Statistical analyses

The data obtained from the item analysis were categorized, tabulated in Excel, and analyzed by SPSS V27 (Armonk, NY: I.B.M. Corp, U.S.A.). Categorical data were presented as frequencies and percentages. The Pearson correlation test measured the correlation between discrimination, difficulty indexes, and distractor efficiency. The significance level was 95%, and any *P* < 0.05 was considered significant.

## Results

The total number of examinees in the final exam of the principle of human diseases was 45. The mean (M), and standard deviation (SD) of the examinees’ age was 20.5 (0.97). The M (SD) of the examinee’s GPA was 3.9 (0.59).

The total number of exam items analyzed was 59 (one item was deleted due to a technical flaw). The exam contained a total of 177 distractors and 59 best answers. The M (SD) of the class score was 40 (5.14). The highest and lowest exam scores achieved by the examinees were 57 and 25, respectively. The KR20 of the exam was 0.91. The M (SD) of DIF was 37.5(19.1), the majority of exam items (72.9%) were of acceptable difficulty, and only two out of 59 were easy (Table 1). The M (SD) of DIS was 0.46 (0.22). The majority of the exam items were excellent in discrimination (69.5%), and only 8 items were non-discriminating (13.6%) (Table 2). Exam items with excellent and good distractor efficiency represent 37.3% each, and only 3.4% (2 out of 59)were of poor efficiency (Table 3).

**Table 1:** Classification of the exam items according to the item’s difficulty index (*n* = 59)

Range	Interpretation	Frequency	Percentage
76–100	Easy	2	3.4
26–75	Acceptable	43	72.9
0–25	Difficult	14	23.7
**Total**		**59**	**100.0**

**Table 2:** Classification of the exam items according to their discrimination index (*n* = 59)

Range	Interpretation	Frequency	Percentage
≤ 0.20	Non discriminating	8	13.6
0.21–0.24	Acceptable	10	16.9
0.25–0.34	Good	0	0
≥ 0.35	Excellent	41	69.5
**Total**		**59**	**100.0**

**Table 3:** Classification of the exam items according to their distractor efficiency (*n* = 59)

NFDs	Interpretation	Score	Frequency	Percentage
3	Poor	0	2	3.4
2	Moderate	33.3	13	22.0
1	Good	66.6	22	37.3
0	Excellent	100	22	37.3
**Total**		**100**	**59**	**100.0**

NFD = Non-functional distractor

The Pearson correlation test shows a significant moderate negative correlation between DE and DIF (*P* = 0.00, *r*=-0.548) and a significant weak negative correlation between DE and DIS (*P* = 0.0476, *r*=- 0.259). A non-significant weak negative correlation was reported between DIF and DIS (Table 4).

Items with excellent distractor efficiency were 22 out of 59; most of the 22 were of acceptable difficulty (90.9%), and 16 had excellent DIS (72.7%). Items with moderate DE were 13 (22%) out of 59, and according to the difficulty index, they were either difficult (53.8%) or acceptable (46.2%). Items with good distractor efficiency out of the 59 were 22; most of them were acceptable (77.3%), and the rest were difficult (22.7%). Items with poor DE were only 2 out of 59, which were difficult and non-discriminating (Table 5).

**Table 4:** The correlation between distractor efficiency, difficulty index, and discrimination index (*n* = 59)

Correlations				
		DE	DIF	DIS
DE	Pearson Correlation	1	-0.548**	− 0.259*
	Sig. (2-tailed)	-	0.0000	0.0476
	N	59	59	59
DIF	Pearson Correlation	-0.548**	1	-0.0492
	Sig. (2-tailed)	0.0000	-	0.7124
	N	59	59	59

**. Correlation is significant at the 0.01 level (2-tailed)

*. Correlation is significant at the 0.05 level (2-tailed)

*DE = distractor efficiency, DIF = difficulty index, DIS = discrimination index*

**Table 5:** shows the items’ distractor efficiency, difficulty index, and discrimination index (*n* = 59)

DE			DIF			DIS		
Interpretation	NU	%	Interpretation	NU	%	Interpretation	NU	%
Excellent	22	37.3	Difficult	0	0.0	Excellent	16	72.7
			Acceptable	20	90.9	Good	0	0.0
			Easy	2	9.1	Acceptable	3	13.6
			-	-	-	None	3	13.6
Moderate	13	22	Difficult	7	53.8	Excellent	8	61.5
			Acceptable	6	46.2	Good	0	0.0
			Easy	0	0.0	Acceptable	4	30.8
			-	-	-	None	1	7.7
Good	22	37.3	Difficult	5	22.7	Excellent	17	77.3
			Acceptable	17	77.3	Good	0	0.0
			Easy	0	0.0	Acceptable	3	13.6
			-	-	-	None	2	9.1
Poor	2	3.4	Difficult	2	100.0	Excellent	0	0.0
			Acceptable	0	0.0	Good	0	0.0
			Easy	0	0.0	Acceptable	0	0.0
			-	-	-	Non	2	100.0

DE = distractor efficiency, DIF = difficulty index, DIS = discrimination index

## Discussion

In the current results, the small standard deviation of students’ GPA and exam scores indicates the data are clustered tightly around the mean. Such results suggested that the student performance is comparable and their exam results are reliable.

The KR-20 of the final course exam was 0.91. KR-20 of 0.91 is ideal for a high-stakes exam, confirms the homogeneity and uni-dimensionality of exam items, and reflects high reliability [3, 36, 37]. Medical education desires values of 0.8 and above for high-stakes exams and lower for in-class assessments. This finding agrees with the earlier work of Kehoe (1995) and Bell (2014) [38, 39]. They reported that exams with more than 50 items should have a KR-20 of 0.8 or more.

The average DIF of the exam was 37.5 and the standard deviation was 19.1, which is an acceptable difficulty. Many studies reported an average difficulty index of exams. The current difficulty index is lower than reported in the previous work of Anathakrishnan (39.4 ± 21.4%), Pande et al. (52.53 ± 20.59), and Karelia et al. (47.17 ± 19.79 to 58.8 ± 19.33) [40–42].

The current study shows that most exam items were of acceptable difficulty (72.9%). The present findings differ from those reported by Sugianto (2020) for an ideal balanced exam, where the percentages of moderate and difficult items in the exam exceed the recommended rates [19]. The difficulty index of an item is related to the item and student performance in the given time. Many causes can be connected to the item’s difficulty, such as uncovered content material, writing flaws, and a wrong key. Despite the difference in the percentages from the ideal difficulty-balanced exam, the average score (40 out of 59) and the class median (33 out of 59) indicate a good performance from students.

The average discrimination of the exam index was 0.46, and the standard deviation was 0.22, which is considered excellent or very discriminating [43, 44]. The low standard deviation of the discrimination index means that the discrimination powers of the items are related, and since they are in the range of excellent or very discriminating, they are reasonably good. Also, this suggestion is supported by the result that about 69.5% of the exam items were categorized as excellent discriminating, and only 13.6% were not discriminating.

The correlation between DE and DIF (*p* = 0.000, *r*= -0.548) indicates that items with efficient distractors (low number of NFD) are associated with those having a low difficulty index (difficult items) and vice versa. The current findings support the previous research on the relationship between DE and DIF. They reported an association between highly efficient items and items with low difficulty index [3, 37, 45, 46]. When all the distractors are functioning, the possibility of eliminating them due to any cause other than knowledge is less. Thus, such items are expected to have acceptable difficulty and good discrimination indexes. Items with a high number of NFDs (low efficiency) can be answered by students more frequently because they can eliminate the NFDs easily. Consequently, such items are expected to be easy rather than difficult items without flaws.

The correlation between DE and DIS is significantly negative (*P* = 0.0476, *r*=-0.259). In such a correlation, items with efficient distractors are associated with low-discriminating items. The current findings support the previous studies of Mitra et al. and Bhat et al. [47, 48]. Contrary to the present results, a positive correlation was reported between DE and DIS [42, 49].

Items with a low discrimination index cannot discriminate between high and lower achievers. In such a case, these items are expected to be easy or difficult, or no students answer them. The presence of easy items can be due to mastering the content material of the item, the repeated use of the item, or technical flaws such as a high number of NFDs [15, 22, 26].

Items with non-functional distractors can be present in any examination [23]. The second step, after defining them in the running examination, remains open and debatable between two options: updating the item distractors for the next use or deleting the item from the current exam. It was reported that items with NFDs should be replaced by more plausible distractors or removed from the test [23, 50]. Kehoe (1995) reported that deleting such items is ethical and justifiable [38]. He asserted that the purpose of the test is to figure out each student’s rank. Using items with unsatisfactory psychometrics goes against this goal, and the accuracy of the ensuing ranking suffers as a result. In the current study, deleting items with three non-functional distractors increased the average DIF from 37.5 ± 19.1 to 38.65 ± 18.07 and the DIS from 0.46 ± 0.22 to 0.47 ± 0.22.

The presence of NFD can be related to decreased training of item constructors, the blueprinting of the exam, and the content material. The selection of distractors is governed by being plausible and conveying a miss concept about correct information. Another issue is the possible number of distractors that can be created or used. Due to the nature of the content material from which the item is being constructed, it is frequently difficult for item constructors to develop three or more plausible distractors with the same quality. In such cases, the additional distractors are often used as fillers [23]. Many researchers reported no difference in the psychometric properties of the exams when using three or five options [23, 51–55]. Thus, reducing the number of distractors can be part of the solution to the NFD issue.

The study findings and the correlation between DE, DIF, and DIS suggest that decreasing the number of NFD or increasing DE can increase the parameters of the item analysis and, consequently, the assessment. Training of training of item constructors and the use of exam blueprinting can improve the DE.

## Conclusion

A significant moderate negative correlation exists between DE and DIF (*P* = 0.00, *r* = -0.548) and a significant weak negative correlation between DE and DIS (*P* = 0.0476, *r* = -0.259). DIF has a non-significant negative correlation with DIS (*P* = 0.7124, *r* = -0.0492). DE impacts both DIF and DIS. Items with efficient distractors (low number of NFD) are associated with those having a low difficulty index (difficult items) and discriminating items. The presence of NFD can be related to decreased training of item constructors, the blueprinting of the exam, and the content material. The authors recommend conducting the study with many courses and a large sample size for more robust and precise results to help understand the relation between DE and the other parameters of item analysis.

### Study limitations

Small sample size.

They are applied in one course and institute.

### Study strength

The study reported significant results.

The study shed light on an important topic.

Study protocol can be applied to studies of large sample sizes.

## Data Availability

The datasets used and analyzed during the current study are available from the corresponding author upon reasonable request.

## Abbreviations

DE: Distractor efficiency
MCQs: Multiple-choice questions
DIF: Difficulty index
DIS: Discrimination index
IA: Item analysis
KR-20: Kuder–Richardson formulas
M: Mean
SD: Standard deviation
FD: Functioning distractor
NFD: Non-functioning distractors

## References

[1] Sahoo DP, Singh R (2017). Item and distracter analysis of multiple choice questions (MCQs) from a preliminary examination of undergraduate medical students. Int J Res Med Sci.

[2] Jaleel A, Khanum Z, Siddiqui IA, Ali M, Khalid S, Khursheed R (2020). Discriminant validity and reliability of scores of multiple choice and short essay questions. Biomedica.

[3] Rezigalla AA. Item analysis: Concept and application. In: *Medical Education for the 21st Century* edn. Edited by Firstenberg MS, Stawicki SP. London: Intechopen; 2022: 1–16.

[4] Salih KEM, Jibo A, Ishaq M, Khan S, Mohammed OA, Al-Shahrani AM, Abbas M (2020). Psychometric analysis of multiple-choice questions in an innovative curriculum in Kingdom of Saudi Arabia. J Family Med Prim care.

[5] Allanson P, Notar C (2019). Writing multiple choice items that are reliable and valid. Am Int J Humanit Social Sci.

[6] Iqbal Z, Saleem K, Arshad HM (2023). Measuring teachers’ knowledge of student assessment: development and validation of an MCQ test. Educational Stud.

[7] Naidoo M (2023). The pearls and pitfalls of setting high-quality multiple choice questions for clinical medicine. South Afr Family Practice: Official J South Afr Acad Family Practice/Primary Care.

[8] Suryono W, Harianto BB (2023). Item analysis of multiple choice questions (MCQs) for dangerous Goods courses in Air Transportation Management Department. Technium Social Sci J.

[9] Uddin ME. Common item violations in multiple choice questions in Bangladeshi recruitment tests. Local Research and Glocal perspectives in English Language Teaching: teaching in changing Times. edn.: Springer; 2023. pp. 377–96.

[10] Kumar AP, Nayak A, Chaitanya KMS, Ghosh K (2023). A Novel Framework for the generation of multiple choice question stems using semantic and machine-learning techniques. Int J Artif Intell Educ.

[11] Yahia AIO (2021). Post-validation item analysis to assess the validity and reliability of multiple-choice questions at a medical college with an innovative curriculum. Natl Med J India.

[12] Rao C, Kishan Prasad H, Sajitha K, Permi H, Shetty J (2016). Item analysis of multiple choice questions: assessing an assessment tool in medical students. Int J Educational Psychol Researches.

[13] Abdulghani HM, Ahmad F, Ponnamperuma GG, Khalil MS, Aldrees A (2014). The relationship between non-functioning distractors and item difficulty of multiple choice questions: a descriptive analysis. J Health Specialties.

[14] Rezigalla AA, Eleragi AME, Ishag M (2020). Comparison between students’ perception toward an examination and item analysis, reliability and validity of the examination. Sudan J Med Sci.

[15] Kumar D, Jaipurkar R, Shekhar A, Sikri G, Srinivas V (2021). Item analysis of multiple choice questions: a quality assurance test for an assessment tool. Med J Armed Forces India.

[16] Warburton B, Conole G. Key findings form recent literature on computer-aided Assessment. In.: ALTC-C University of Southampton; 2003. pp. 1–19.

[17] Mhairi M, Hesketh I. Multiple response questions–allowing for chance in authentic assessments. In: *7th International CAA Conference* Edited by J C. Loughborough:Loughborough University; 2003.

[18] Licona-Chávez AL, Velázquez-Liaño LR (2020). Quality assessment of a multiple choice test through psychometric properties. MedEdPublish.

[19] Sugianto A (2020). Item analysis of English summative test: Efl teacher-made test. Indonesian EFL Res Practices.

[20] Considine J, Botti M, Thomas S (2005). Design, format, validity and reliability of multiple choice questions for use in nursing research and education. Collegian.

[21] Haladyna TM, Rodriguez MC (2021). Using full-information item analysis to Improve Item Quality. Educational Assess.

[22] Obon AM, Rey KAM. Analysis of Multiple-Choice Questions (MCQs): Item and test statistics from the 2nd year nursing qualifying exam in a University in Cavite, Philippines. In: *Abstract Proceedings International Scholars Conference*: 2019; 2019: 499–511.

[23] Tarrant M, Ware J, Mohammed AM (2009). An assessment of functioning and non-functioning distractors in multiple-choice questions: a descriptive analysis. BMC Med Educ.

[24] Mahjabeen W, Alam S, Hassan U, Zafar T, Butt R, Konain S, Rizvi M (2017). Difficulty index, discrimination index and distractor efficiency in multiple choice questions. Annals PIMS-Shaheed Zulfiqar Ali Bhutto Med Univ.

[25] Abdalla ME (2011). What does item analysis tell us? Factors affecting the reliability of multiple choice questions (mcqs). Gezira J Health Sci.

[26] Fozzard N, Pearson A, du Toit E, Naug H, Wen W, Peak IR (2018). Analysis of MCQ and distractor use in a large first year Health Faculty Foundation Program: assessing the effects of changing from five to four options. BMC Med Educ.

[27] Abdellatif H, Al-Shahrani AM (2019). Effect of blueprinting methods on test difficulty, discrimination, and reliability indices: cross-sectional study in an integrated learning program. Adv Med Educ Pract.

[28] Rejeki S, Sari ABP, Sutanto S, Iswahyuni D, Yogyanti DW, Anggia H (2023). Discrimination index, difficulty index, and distractor efficiency in MCQs English for academic purposes midterm test. J Engl Lang Pedagogy.

[29] Licona-Chávez AL, Velázquez-Liaño LR (2020). Quality assessment of a multiple choice test through psychometric properties. MedEdPublish.

[30] McCrossan P, Nicholson A, McCallion N (2022). Minimum accepted competency examination: test item analysis. BMC Med Educ.

[31] Burud I, Nagandla K, Agarwal P (2019). Impact of distractors in item analysis of multiple choice questions. Int J Res Med Sci.

[32] Rezigalla AA (2020). Observational study designs: Synopsis for selecting an appropriate Study Design. Cureus.

[33] Elgadal AH, Mariod AA (2021). Item analysis of multiple-choice questions (MCQs): Assessment Tool for Quality Assurance measures. Sudan J Med Sci.

[34] Triono D, Sarno R, Sungkono KR. Item Analysis for Examination Test in the Postgraduate Student’s Selection with Classical Test Theory and Rasch Measurement Model. 2020 International Seminar on Application for Technology of Information and Communication (iSemantic): 2020. IEEE; 2020. pp. 523–9.

[35] Date AP, Borkar AS, Badwaik RT, Siddiqui RA, Shende TR, Dashputra AV (2019). Item analysis as tool to validate multiple choice question bank in pharmacology. Int J Basic Clin Pharmacol.

[36] Shahid R, Zeb S, Hayat U, Yasmeen S, Khalid M (2021). Item analysis of Pathology Assessment of 4th year MBBS at Rawalpindi Medical University Pakistan. J Comm Med Pub Health Rep.

[37] Chauhan GR, Chauhan BR, Vaza JV, Chauhan PR, Chauhan B, Vaza J, CHAUHAN PR (2023). Relations of the number of functioning distractors with the Item Difficulty Index and the item discrimination power in the multiple choice questions. Cureus.

[38] Kehoe J (1995). Basic item analysis for multiple-choice tests. Practical Assess Res Evaluation.

[39] Bell BA. Pretest–Posttest Design. In: *Encyclopedia of research design. Volume 2*, edn. Edited by Salkind NJ. Thousand Oaks: SAGE Publications, Inc.; 2014: 1087–1092.

[40] Anathakrishnan N. The item analysis. In: *Medical education principles and practice. Volume 2*, edn. Edited by Anathakrishnan N, Sethukumaran K, Kumar S. Pondicherry, India: JIPMER; 2000: 131–137.

[41] Karelia BN, Pillai A (2013). The levels of difficulty and discrimination indices and relationship between them in four-response type multiple choice questions of pharmacology summative tests of year II M.B.B.S students. Int E-J Sci Med Educ.

[42] Pande SS, Pande SR, Parate VR, Nikam AP, Agrekar SH (2013). Correlation between difficulty and discrimination indices of MCQs in formative exam in physiology. South-East Asian J Med Educ.

[43] Abdulghani HM, Ahmad F, Ponnamperuma GG, Khalil MS, Aldrees A (2014). The relationship between non-functioning distractors and item difficulty of multiple choice questions: a descriptive analysis. J Health Specialties.

[44] Aljehani DK, Pullishery F, Osman OAE, Abuzenada BM (2020). Relationship of text length of multiple-choice questions on item psychometric properties–A retrospective study. Saudi J Health Sci.

[45] Alareifi RM (2023). Analysis of MCQs in summative exam in English: Difficulty Index, discrimination index and relationship between them. J Eduction Hum Sci.

[46] Chit YZ, Aung AA. An Analysis on Functioning and Non Functioning Distractors in Physics Multiple Choice Question. In: *INTERNATIONAL ASIAN CONGRESS ON CONTEMPORARY SCIENCES-IV 2020; Baku, Azerbaijan* 2020: 218–227.

[47] Bhat SK, Prasad KHL (2021). Item analysis and optimizing multiple-choice questions for a viable question bank in ophthalmology: a cross-sectional study. Indian J Ophthalmol.

[48] Mitra N, Nagaraja H, Ponnudurai G, Judson J (2009). The levels of difficulty and discrimination indices in type a multiple choice questions of pre-clinical semester 1 multidisciplinary summative tests. Int E-J Sci Med Educ.

[49] Kheyami D, Jaradat A, Al-Shibani T, Ali FA (2018). Item analysis of multiple choice questions at the department of paediatrics, Arabian Gulf University, Manama, Bahrain. Sultan Qaboos Univ Med J.

[50] Haladyna TM, Downing SM (1989). Validity of a taxonomy of multiple-choice item-writing rules. Appl Measur Educ.

[51] Vyas R, Supe A (2008). Multiple choice questions: a literature review on the optimal number of options. Natl Med J India.

[52] Kanzow AF, Schmidt D, Kanzow P (2023). Scoring single-response multiple-choice items: scoping review and comparison of different scoring methods. JMIR Med Educ.

[53] Landrum RE, Cashin JR, Theis KS (1993). More evidence in favor of three-option multiple-choice tests. Educ Psychol Meas.

[54] Owen SV, Froman RD (1987). What’s wrong with three-option multiple choice items?. Educ Psychol Meas.

[55] Shizuka T, Takeuchi O, Yashima T, Yoshizawa K (2006). A comparison of three-and four-option English tests for university entrance selection purposes in Japan. Lang Test.

